# Adebrelimab plus chemotherapy vs. chemotherapy for treatment of extensive-stage small-cell lung cancer from the US and Chinese healthcare sector perspectives: a cost-effectiveness analysis to inform drug pricing

**DOI:** 10.3389/fphar.2023.1241130

**Published:** 2023-07-20

**Authors:** Yena Gan, Fenghao Shi, He Zhu, Sheng Han, Duoduo Li

**Affiliations:** ^1^ Dongzhimen Hospital, Beijing University of Chinese Medicine, Beijing, China; ^2^ International Research Center for Medicinal Administration, Peking University, Beijing, China; ^3^ School of Pharmaceutical Sciences, Peking University, Beijing, China

**Keywords:** ES-SCLC, adebrelimab, healthcare, cost-effectiveness, drug pricing

## Abstract

**Purpose:** The aim of this study was to evaluate the cost-effectiveness of a recently approved first-line therapy (adebrelimab plus chemotherapy vs. chemotherapy alone) for patients with extensive-stage small-cell lung cancer (ES-SCLC) in the US and China, and to estimate the reasonable range of adebrelimab price from the decision-makers.

**Methods:** Several partitioned survival models were built to compare the cost and effectiveness of adebrelimab plus chemotherapy vs. chemotherapy alone over a 10-year time horizon. Clinical efficacy and safety data were extracted from the CAPSTONE-1 trial. Costs and utilities were obtained from previously published studies. Sensitivity, scenario and subgroup analyses were performed to explore the uncertainty of the model outcomes. Price simulation was conducted at three thresholds of willingness-to-pay (WTP), including WTP of $100,000 in the US and of $37,422 in China, 0.5WTP of $50,000 in the US and of $18,711 in China, and 1.5WTP of 150,000 in the US and of $56,133 in China.

**Findings:** Base-case analysis at $1382.82/600 mg of adebrelimab price indicated that adebrelimab plus chemotherapy would be cost-effective in the US at the WTP threshold of $100,000, but not in China at the WTP threshold of $37,422. If PAP was taken into account, the regimen would be cost-effective in China at the given WTP. The results of price simulation indicated that adebrelimab plus chemotherapy was completely favored in the US if adebrelimab price was less than $8894.98/600 mg (total quality-adjusted life years [QALYs] were calculated with progression-based utility [PB-utility]) or $8912.51/600 mg (total QALYs were calculated with time-to-death utility [TTD-utility]) at the WTP threshold of $100,000; if adebrelimab price was reduced by at least $202.03/600 mg (total QALYs were calculated with PB-utility) or $103.06/600 mg (total QALYs were calculated with TTD-utility), the regimen was also cost-effective in China without PAP at the WTP threshold of $37,422. The above results were stable in the sensitivity analyses. Subgroup analysis found that the subgroup with better survival benefits tended to have a higher probability of cost-effectiveness, which was also associated with adebrelimab price.

**Implications:** First-line adebrelimab plus chemotherapy represented a dominant treatment strategy comparing with chemotherapy alone in the US and also did in China with PAP at $1382.82/600 mg of adebrelimab price. Decision-makers could benefit from pricing strategy provided by this study in making optimal decisions. More evidences were needed to verify and improve the results.

## 1 Introduction

Lung cancer is a malignancy with the highest incidence and mortality worldwide, and small-cell lung cancer (SCLC) is its most aggressive type, nearly accounting for 13% (Howlader et al., 2020). SCLC is the leading cause of death for men and the second for women (Siegel et al., 2021). Without treatment, its overall survival (OS) is only 2–4 months and 5-year survival rates between 5% and 10% (PDQ Adult Treatment Editorial Board, 2002; van Meerbeeck et al., 2011). Approximately 80%–85% of SCLC are progressed to extended-stage disease (ES-SCLC) when first diagnosis (Schwendenwein et al., 2021). For more than 3 decades, etoposide with platinum (carboplatin or cisplatin) has been the standard first-line treatment of ES-SCLC, significantly improving the 5-year survival rate of patients (6%–7%) (Simon et al., 2003). However, most patients rarely have a long-term survival, with a median OS (mOS) of 10 months (Davies et al., 2004). It is urgently needed to explore more effective first-line treatment to improve the clinical prognosis and outcomes of ES-SCLC.

The development of immune checkpoint inhibitors (ICIs) has broken the traditional treatment layout for ES-SCLC (Lee et al., 2022). ICIs could reduce immunosuppression in the tumor microenvironment by inhibiting cytotoxic T-lymphocyte antigen 4 (CTLA-4) and programmed cell death-1/programmed cell death receptor ligand-1 (PD-1/PD-L1) pathway, and reactivate the anti-tumor function of the immune system (Munn and Bronte, 2016), showing promising anti-tumor activity to ES-SCLC with high tumor mutational burden (TMB) and PD-L1 expression (Cortellini, 2020). Published literature revealed that OS was significantly prolonged in patients with ICIs plus chemotherapy than chemotherapy or ICIs alone (Arriola et al., 2022; Yu et al., 2023). In 2021, guidelines published by the NCCN (National Comprehensive Cancer Network) and CSCO (Chinese Society of Clinical Oncology) formally introduced ICIs (atezolizumab or durvalumab) plus chemotherapy as first-line treatment of ES-SCLC based on IMpower133 and CASPIAN trials (Ganti et al., 2021).

Adebrelimab (SHR-1316), a recombinant fully humanized IgG4 monoclonal antibody with high affinity and specificity for PD-L1, has already been approved for anti-cancer treatment in China by Centre for Drug Evaluation (CDE) in 2022. This approval is based on the results from CAPSTONE-1 (a double-blind, placebo-controlled, phase-III trial done in 47 tertiary hospitals in China; NCT03711305), in which the first-line treatment adebrelimab plus chemotherapy (etoposide-carboplatin) vs. chemotherapy alone provided significant clinical benefits and similar safety in patients with previously untreated ES-SCLC (Wang et al., 2022). For the fist-line treatment of ES-SCLC, the reduced risk of 33% for progression or death in the CAPSTONE-1 with adebrelimab plus chemotherapy was similar to the IMpower133 with atezolizumab plus chemotherapy (23%) and CASPIAN with durvalumab plus chemotherapy (22%) (Horn et al., 2018; Paz-Ares et al., 2019; Wang et al., 2022). Previous studies revealed that neither atezolizumab plus etoposide-carboplatin nor durvalumab plus etoposide-platinum was a cost-effective fist-line treatment of ES-SCLC from the perspective of the US or China (Li et al., 2019; Zhou et al., 2019; Ionova et al., 2022; Liu and Kang, 2022). In addition, among other first-line treatment for ES-SCLC (pembrolizumab, nivolumab, ipilimumab, serplulimab), serplulimab plus chemotherapy is likely to be cost-effective only in China. From the US perspective, no cost-effective treatment has been identified (Kang et al., 2021; Liu et al., 2021; Zhu et al., 2021; Shao et al., 2023).

As a novel and potent regimen, adebrelimab plus chemotherapy presented comparable clinical benefits, but no relevant study reported its weight against financial burden. Due to the rising medical expenditures and the increasing population, decision-makers are required to assess the cost-effectiveness of adebrelimab plus chemotherapy as a first-line treatment for previously untreated ES-SCLC. Additionally, evaluating the reasonable range of adebrelimab price is necessary. Therefore, the study aimed to estimate these from the perspectives of healthcare sector in the US and China.

## 2 Methods

This economic evaluation followed the Consolidated Health Economic Evaluation Reporting Standards (CHEERS) reporting guideline (Husereau et al., 2013) and based on the CAPSTONE-1 trial (Wang et al., 2022), publicly available databases and published literature, using no individual patient-level data to inform the model, so it was exempted from the approval of the institutional research ethics board.

### 2.1 Model structure

**FIGURE 1:**
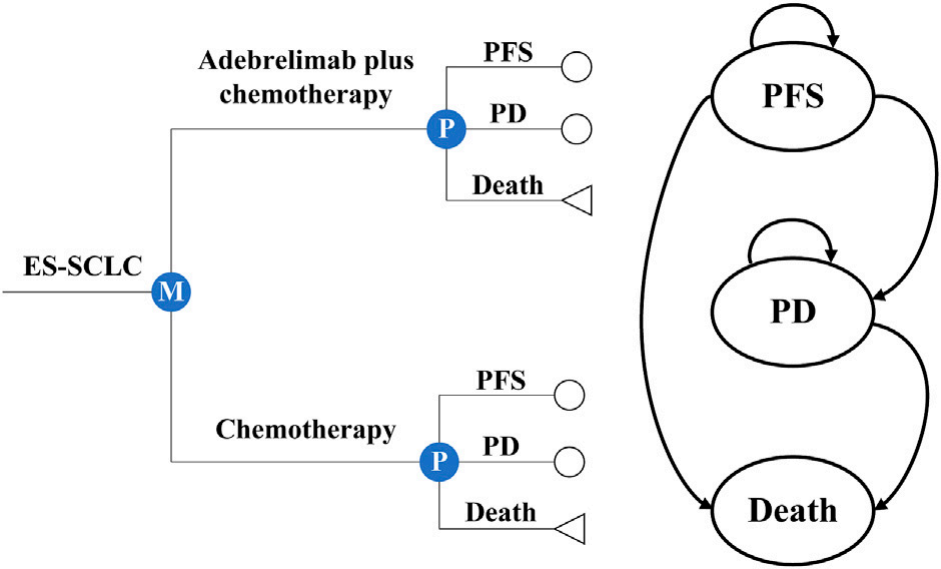
Partitioned survival models and decision tree with three health states. Circles were used to represent the survival status, including progression-free survival (PFS) and progressive disease (PD), while triangles were used to represent the deceased status.

Based on the CAPSTONE-1 trial, we established the partitioned survival models combining with a decision tree to assess the costs and effectiveness of adebrelimab plus chemotherapy vs. chemotherapy alone as first-line therapy for previously untreated ES-SCLC (Wang et al., 2022) (Figure 1). In the models, three mutually exclusive health states were included to represent the progression of ES-SCLC: PFS, progressive disease (PD), and death. All patients started from PFS and were treated with chemotherapy alone or plus adebrelimab until disease progression, or unacceptable toxicity, or up to 2 years of treatment, whichever occurred first (eMethods 1 in the Supplementary material S1). Per the CAPSTONE-1 protocol, patients in the hypothetical cohort could receive subsequent therapies after discontinued adebrelimab or placebo (eMethods 2 and 3 in the Supplementary material S1).

The model cycle length was a 3-week treatment cycle and the models were run for 10 years expected to include patients’ entire life span (Saltos et al., 2020). The primary outcomes included total costs, life-years (LYs), quality-adjusted life years (QALYs), discounted at an annual rate of 3% for the US(Su et al., 2021) and of 5% for China (Yue et al., 2021). Incremental cost-effectiveness ratios (ICERs) were also estimated to present the incremental costs of acquiring an extra unit of QALY and were compared with a willingness-to-pay (WTP) threshold of $37,422 (three times Chinese gross domestic product [GDP] *per capita*) for China (National Bureau of Statistics, 2022) and of $100,000 for the US (Neumann et al., 2014). Adebrelimab plus chemotherapy could be regarded as cost-effective comparing with chemotherapy alone if the ICER was less than the threshold, otherwise, chemotherapy was more economical. The model was constructed *via* R (version 4.1.2, flexsurv and survHE packages) and Excel spreadsheet software (version 16, Microsoft).

### 2.2 Model probabilities

The probabilities for the partitioned states were determined by Kaplan-Meier (K-M) curves of OS and PFS from the CAPSTONE-1 trial (Wang et al., 2022). First, we extracted and reconstructed the individual patient data (IPD) basing on K-M curves (Guyot et al., 2012). Then, a range of commonly used parametric survival models were used to fit the IPD data, including the weibull, exponential, gompertz, log-logistic, log-normal, gamma, generalized gamma, fractional polynomial (FP) (Royston and Sauerbrei, 2005), restricted cubic spline (RCS) (Rutherford et al., 2015) and royston-parmar spline (RP) models (Neumann et al., 2014). According to statistical goodness-of-fit basing on akaike information criterion (AIC), extrapolation performance basing on log likelihood, clinical rationality basing on mean squared errors (MSE), and visual inspection, we evaluated the fitting degree of the alternative models and selected the best one to extrapolate the K-M curves beyond the follow-up duration of the CAPSTONE-1 trial (Latimer, 2013) (eMethods 4, Supplementary Table S3; Supplementary Figure S1).

### 2.3 Cost estimates

Only direct medical costs were considered in 2023 US dollars as follows: regimen related costs (hypothesizing no medicine wastage), costs of serious treatment-related adverse events (TRAEs, assuming that serious TRAEs appeared only the first cycle in the PFS and PD states), and costs of follow-up (eMethods 3 and Supplementary Table S4). Regimen related costs involved drug acquisition and administration costs. According to the relative dose intensity (delivered total dose/planned total dose per protocol for treatment period) reported in the CAPSTONE-1, the total delivered dose of each drug used was basically consistent with the total planned dose per protocol (Supplementary Table S2). Therefore, it is assumed that treatment period and dose intensity of each drug in the cohort correspond to the median cycle number and average delivered dose intensity reported in the trial (model 1) (Supplementary Table S1). We also made an additional assumption based on the CAPSTONE-1 protocol, which stated that patients would continue to receive adebrelimab or placebo for 2-year if their disease did not progress or they did not experience intolerable toxicity (model 2), where the dose intensity of each drug was consistent with the model 1 (eMethods 3 in the Supplementary Mateial S1).

The costs of serious TRAEs in the models by multiplying the incidences of serious TRAEs by the cost of individual event treatment. All costs were derived from the CAPSTONE-1 trial, the publicly available databases (e.g., Centers for Medicare and Medicaid Services (CMS) reimbursement schedule (U.S. Centers for Medicare & Medicaid Services, 2023), Average Wholesale Price (AWP) (UpToDate, 2023), Yaozhi data (Yaoch, 2023)) and previously published studies.

### 2.4 Health utilities

We used utility to measure patients’ health-related quality of life (QoL) at a particular health state, which is often evaluated by three-level EuroQoL-5D (EQ-5D) questionnaire and is referred to as QALYs (0 for death and 1 for perfect heath). However, the CAPSTONE-1 trial did not report it, we assigned the utilities of 0.70, 0.60 and 0 for the PFS, PD and death states, respectively, according to the previous publication (Them were referred to as “progression-based utility (PB-utility)") (Vedadi et al., 2021). A study on the health utility for ES-SCLC patients proposed time-to-death utility (TTD-utility) based on patient survival after diagnosis (National Institute for Health and Care Excellence, 2020). Therefore, we also included TTD-utility in the scenario analysis. In addition, the disutility caused by TRAEs were considered. In view of grade 1/2 TRAEs were manageable and the correlation with QoL was low, we included only serious TRAEs (grade≥3, a frequency of greater than 5%). Thus, utilities were modulated by subtracting the product of multiplying incidences by corresponding disutilities of serious TRAEs. In the CAPSTONE-1 trial, both groups occurred haematological adverse events, including decreased neutrophil count (76% in the adebrelimab group and 75% in the chemotherapy group), decreased white blood cell count (46% vs. 38%), decreased platelet count (38% vs. 34%), and anaemia (28% vs. 28%) (Wang et al., 2022).

### 2.5 Price simulation

There is currently no information available on the adebrelimab price in the US, so we assumed it to be $X/600 mg (average price), where X is 1382.82 in the base-case analysis. However, it is only the current adebrelimab price that has been approved for sale in China. Whether it could make adebrelimab plus chemotherapy an economic choice under the healthcare system is not known, and it might not be applicable in the US. Therefore, we considered the upper limit of adebrelimab price ($Xmax/600 mg) based on a 50% fluctuation range around the WTP (from 0.5WTP to 1.5WTP). When the value of X is lower than Xmax, it is likely that adefrelimab plus chemotherapy will become an economic choice.

### 2.6 Sensitivity analysis

We used a series of sensitivity analyses to predict the uncertainty of the model outcomes. One-way sensitivity analysis (OWSA) was conducted to examine the individual effects of a certain parameter on the ICERs according to varied values of this parameter within its preset plausible range collected from 95% confidence intervals or assumed as ± 20% variations of the baseline values, parameters with high uncertainty were considered to have ±30% variations of the baseline values. Although adebrelimab price might have an impact on the model outcomes, its estimation was based on a comprehensive consideration of other model factors and cannot be evaluated as an independent factor, thus it was not included in the OWSA. The results were presented in the tornado diagrams. We also conducted probabilistic sensitivity analysis (PSA) to assess the synthetical influence of multiple parameters by jointly sampling the key model parameters from the pre-specified statistical distribution and then performing 10,000 Monte Carlo simulations. According to the recommendations of the International Society for Pharmacoeconomics and Outcomes Research-Society for Medical Decision Making (ISPOR-SMDM) Modeling Good Research Practice Working Group, gamma distribution was selected for costs, and beta distribution for proportions, incidences, and utilities (Su et al., 2021; Shao et al., 2023). The simulation results were presented in the scatter plots of incremental benefits and costs (Briggs et al., 2012) (Supplementary Table S3). Supplementary Table S4 detailed the baseline values, ranges, and distributions of model parameters in the sensitivity analyses.

### 2.7 Scenario analysis

In scenario 1, we used TTD-utility to calculate the health benefits for patients in the CAPSTONE-1 and compared the results with those obtained using PB-utility. This was used to evaluate the impact of health utility from different estimating methods on economic evaluation of treatment. Additionally, from the Chinese perspective, we also considered the impact of patient assistance program (PAP) on the economic evaluation of treatment in the scenario 2 (Lu et al., 2018).

### 2.8 Subgroup analysis

The CAPSTONE-1 reported HRs of OS and PFS for patient with different characteristics (Wang et al., 2022). Thus, we calculated survival data and evaluated the cost-effectiveness of adebrelimab plus chemotherapy in several subgroups with different characteristics (age, sex, Eastern Cooperative Oncology Group (ECOG) performance status score, smoking history, lactate dehydrogenase (LDH) concentrations at enrolment, status of liver metastases, status of brain metastases, disease stage, and PD-L1 tumor proportion score). When calculating the cost-effectiveness results for a certain characteristic, we assumed that the other characteristics of this subgroup were consistent with the overall population in the base-case. The chi-squared and Fisher exact tests were used to assess the consistency of PSA results between two subgroups. *p*-value <0.05 was considered statistically significant.

## 3 Results

### 3.1 Base-case analysis

The base-case analysis found that when the adebrelimab price was set at $1382.82/600mg, adebrelimab plus chemotherapy with an ICER of $43444.93/QALY was not an economic choice in China, but it had an absolute economic advantage in the US with the costs saving of $183433.14 and incremental QALYs of 0.37, which were not affected by the assumptions of treatment duration (Table 1). For the PFS state, the economic conclusion of adebrelimab plus chemotherapy from the Chinese perspective was consistent with it for the OS state ($53661.92/QALY in model 1 and $103604.95/QALY in model 2); adebrelimab plus chemotherapy was only economic in model 1 with an ICER of $59858.96/QALY from the US perspective (Supplementary Table S5).

**TABLE 1:** Results of base-case analysis. Model 1 assumed that treatment period and dose intensity of each drug in the cohort correspond to the median cycle number and average delivered dose intensity reported in the trial. Model 2 stated that patients would continue to receive adebrelimab or placebo for 2-year if their disease did not progress or they did not experience intolerable toxicity.

Analysis Perspective			Costs, $	LYs	QALYs	Incremental Costs, $	Incremental QALYs	ICER, $/QALY
**China**	**Model 1**	Chemotherapy group	19698.74	1.32	0.82			
		Adebrelimab group	34976.93	1.89	1.17	15278.19	0.35	43444.93
	**Model 2**	Chemotherapy group	20173.69	1.32	0.82			
		Adebrelimab group	50275.52	1.89	1.17	30101.83	0.35	85597.33
**US**	**Model 1**	Chemotherapy group	1267185.73	1.32	0.83			
		Adebrelimab group	1083752.59	1.89	1.20	−183433.14	0.37	-
	**Model 2**	Chemotherapy group	1267421.80	1.32	0.83			
		Adebrelimab group	1099782.13	1.89	1.20	−167639.66	0.37	-

LYs, life-years; QALY, quality-adjusted life year; ICER, incremental cost-effectiveness ratio.

### 3.2 Scenario analysis

The health benefits related to PB-utility was lower than these related to TTD-utility (0.35 QALYs of base-case vs. 0.38 QALYs of scenario 1 in China, 0.37 QALYs of base-case vs. 0.39 QALYs of scenario 1 in the US), which resulted lower ICERs from the Chinese perspective ($43444.93/QALY vs. $40481.74/QALY in model 1, $85597.33/QALY vs. $79759.11/QALY in model 2) (Supplementary Table S6). However, these results did not change the conclusions of the base-case analysis. From the Chinese perspective, adebrelimab plus chemotherapy was an economic option in scenario 2, no matter what assumptions of treatment duration and utility-related methods (Supplementary Table S7). The results of only PFS state also showed the same conclusions.

### 3.3 Sensitivity analysis

**FIGURE 2:**
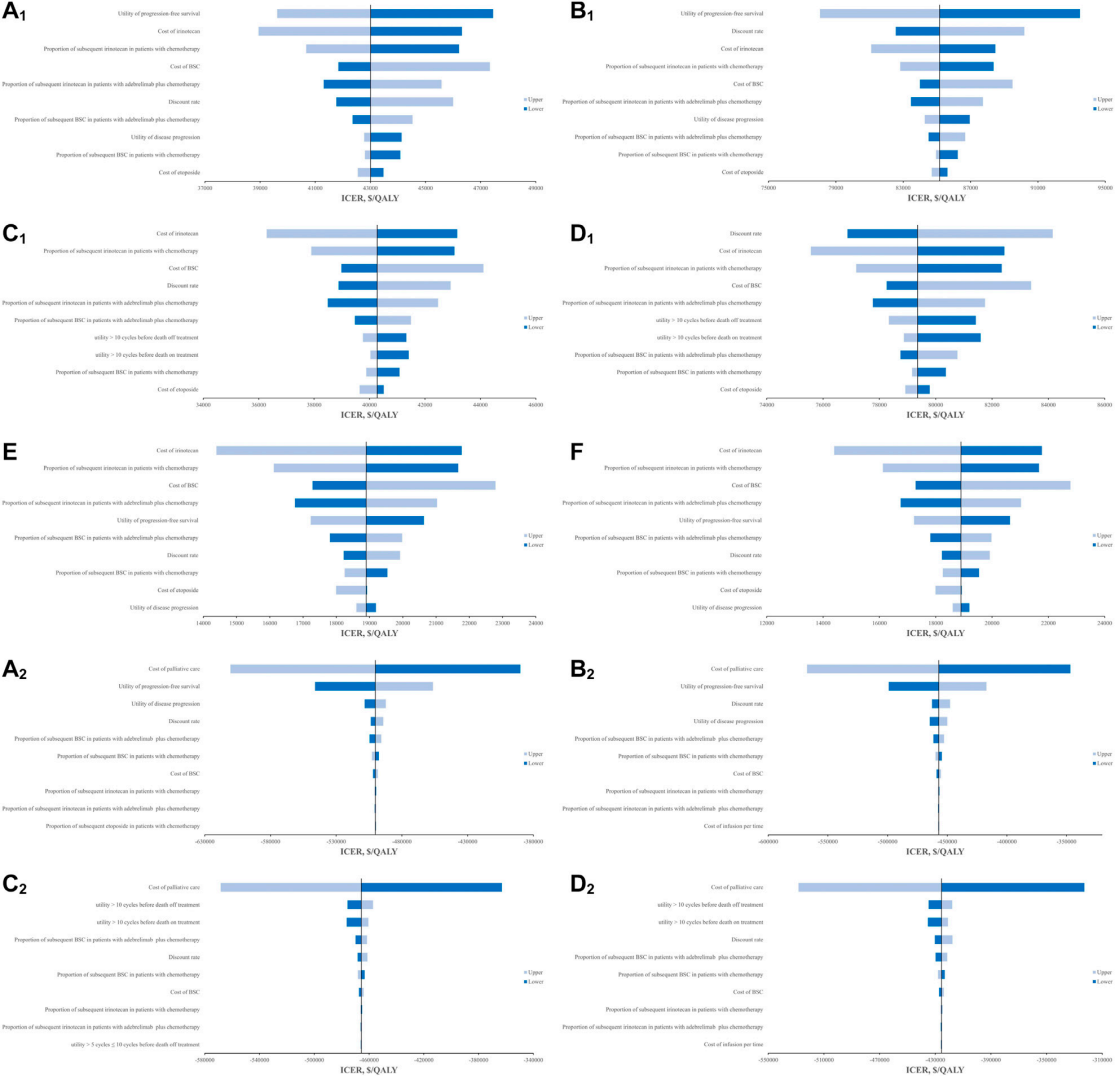
Results of OWSA in the tornado diagrams. **(A**_**1**_ **)** Results of OWSA in model 1 for base-case from Chinese perspective **(B**_**1**_ **)** Results of OWSA in model 2 for base-case from Chinese perspective, **(C**_**1**_ **)** Results of OWSA in model 1 for scenario 1 from Chinese perspective, **(D**_**1**_ **)** Results of OWSA in model 2 for scenario 1 from Chinese perspective, **(E)** Results of OWSA in model 1 for scenario 2 from Chinese perspective, **(F)** Results of OWSA in model 2 for scenario 2 from Chinese perspective. **(A**_**2**_ **)** Results of OWSA in model 1 for base-case from the US perspective, **(B**_**2**_ **)** Results of OWSA in model 2 for base-case from the US perspective, **(C**_**2**_ **)** Results of OWSA in model 1 for scenario 1 from the US perspective, **(D**_**2**_ **)** Results of OWSA in model 2 for scenario 1 from the US perspective.

The OWSA found that the cost of best supportive care (BSC), the proportions of subsequent irinotecan and BSC in both groups, the discount rate, and the utilities including utilities of PFS and PD in base-case and utility over 10 cycles before death in scenario 1 were the main factors affecting the cost-effectiveness analysis results, regardless of the scenarios, treatment duration assumptions, or analysis perspectives (Figure 2). However, they cannot change the economics of adebrelimab plus chemotherapy. From the Chinese perspective, the costs of irinotecan and etoposide were also major factors that affected the results. In scenario 1, when the cost of irinotecan reached the upper value, adebrelimab plus chemotherapy had ICERs below the WTP threshold of $37,422/QALY in model 1. From the US perspective, the cost-effectiveness analysis results were also affected by the costs of palliative care and infusion, and the proportion of subsequent etoposide in chemotherapy group. The results of PSA found that from the Chinese perspective, in order to ensure adebrelimab plus chemotherapy was likely to be an economic option comparing with chemotherapy, the WTP thresholds for models in base-case should respectively be greater than $42,130/QALY and $83,800/QALY, and in scenario 1 should respectively be greater than $39,570/QALY and $78,450/QALY (Supplementary Figure S2).

### 3.4 Price simulation

**FIGURE 3:**
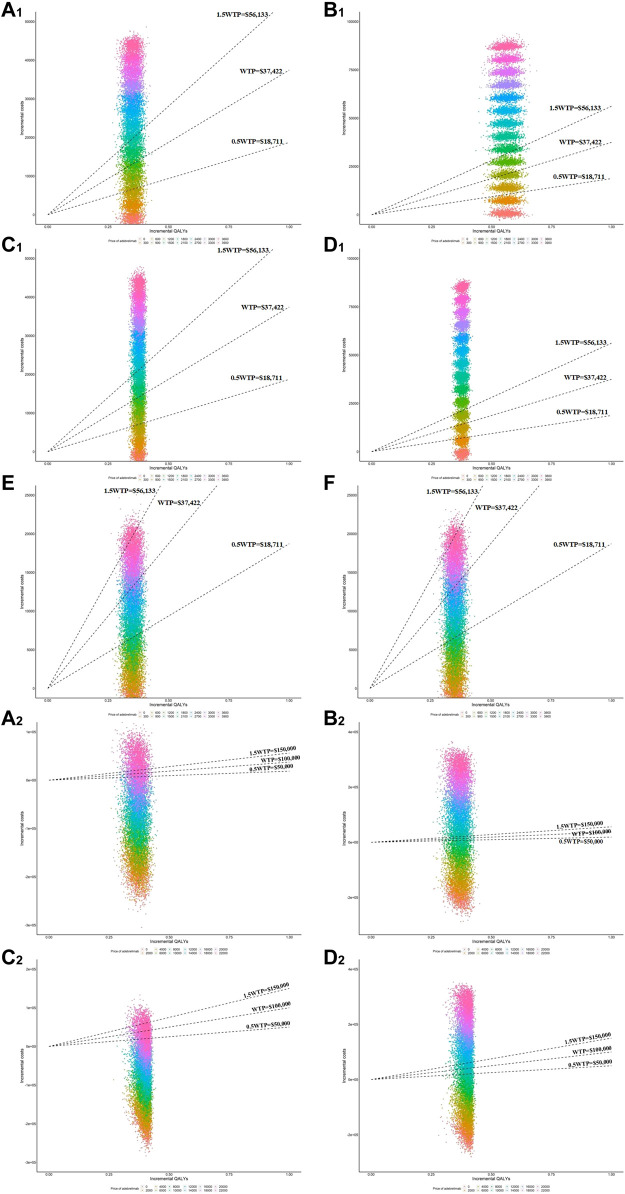
Scatter plots of incremental QALYs and costs in the PSA at various adebrelimab prices. **(A_1_)** Results of PSA in model 1 for base-case from Chinese perspective **(B_1_)** Results of PSA in model 2 for base-case from Chinese perspective, **(C_1_)** Results of PSA in model 1 for scenario 1 from Chinese perspective **(D_1_)** Results of PSA in model 2 for scenario 1 from Chinese perspective, **(E)** Results of PSA in model 1 for scenario 2 from Chinese perspective **(F)** Results of OWSA in model 2 for scenario 2 from Chinese perspective. **(A_2_)** Results of PSA in model 1 for base-case from the US perspective **(B_2_)** Results of PSA in model 2 for base-case from the US perspective, **(C_2_)** Results of PSA in model 1 for scenario 1 from the US perspective **(D_2_)** Results of PSA in model 2 for scenario 1 from the US perspective.

When simply considering the impact of adebrelimab price on the cost-effectiveness results, the estimated Xmax basing on TTD-utility was higher than that basing on PB-utility (Table 2). The implementation of PAP in China produced higher Xmax. We also used PSA to evaluate the cost-effectiveness probability of adebrelimab plus chemotherapy at Xmax (Figure 3). The results of PSA showed that from the US perspective, the estimated Xmax did not always place adebrelimab plus chemotherapy in a relative economic advantage comparing with chemotherapy (the probability of adebrelimab plus chemotherapy being cost-effective is over 50%). However, the differences between these probabilities and 50% of critical probability were very small.

**TABLE 2:** The upper limit of adebrelimab price. PB-utility, progression-based utility; TTD-utility, time-to-death utility. Model 1 assumed that treatment period and dose intensity of each drug in the cohort correspond to the median cycle number and average delivered dose intensity reported in the trial. Model 2 stated that patients would continue to receive adebrelimab or placebo for 2-year if their disease did not progress or they did not experience intolerable toxicity.

Analysis Perspective			0.5WTP, $(%)^a^	WTP, $(%)^a^	1.5WTP, $(%)^a^	ICER = 0, $(%)^b^
**China**	**PB-utility**	**Model 1**	603.74 (70.50%)	1180.79 (68.40%)	1757.85 (63.90%)	26.68 (100.00%)
		**Model 2**	302.47 (98.40%)	604.95 (99.80%)	907.42 (100.00%)	0.00 (100.00%)
	**TTD-utility**	**Model 1**	662.99 (66.50%)	1279.76 (64.50%)	1896.53 (65.10%)	46.22 (100.00%)
		**Model 2**	324.26 (77.50%)	648.53 (73.30%)	972.79 (68.90%)	0.00 (100.00%)
	**PAP**	**Model 1**	1405.42 (60.90%)	2616.65 (62.10%)	3827.87 (68.80%)	194.20 (100.00%)
		**Model 2**	1405.42 (53.70%)	2616.65 (63.40%)	3827.87 (68.60%)	194.20 (100.00%)
**US**	**PB-utility**	**Model 1**	17993.10 (65.20%)	18592.00 (80.70%)	19191.00 (90.70%)	17394.10 (94.10%)
		**Model 2**	9715.03 (48.90%)	10535.10 (76.30%)	11355.10 (50.60%)	8894.98 (94.60%)
	**TTD-utility**	**Model 1**	19112.90 (49.50%)	20832.10 (50.30%)	22551.30 (51.40%)	17393.80 (95.00%)
		**Model 2**	9797.17 (48.30%)	10681.80 (48.10%)	11566.50 (50.50%)	8912.51 (94.80%)

^a^ The adebrelimab price was obtained from OWSA, and the cost-effectiveness probability of adebrelimab plus chemotherapy at the given WTP threshold range was obtained from PSA.

^b^ The results were estimated at the WTP threshold of $37,422/QALY in China and of $100,000/QALY in the US.

From the Chinese perspective, in order for adebrelimab plus chemotherapy to be an economic choice without PAP at the WTP threshold, the Xmax in base-case needed to be reduced by at least 202.03 (1382.82 vs. 1180.79 in model 1, 1382.82 vs. 604.95 in model 2), and in scenario 1 needed to be reduced by at least 103.06 (1382.82 vs. 1279.76 in model 1, 1382.82 vs. 648.53 in model 2). From the US perspective, when the Xmax in base-case was over 8894.98 (model 2) or in scenario 1 was over 8912.51 (model 2), adebrelimab plus chemotherapy was no longer in absolute economic advantage (the incremental costs of adebrelimab plus chemotherapy comparing with chemotherapy was greater than 0).

The treatment costs per cycle of PD-L1/PD-1 drugs simultaneously approved in the US and China for the treatment of lung cancer were shown in Supplementary Table S8). Among these drugs, durvalumab had the closest dosage per cycle to adebrelimab (1500mg/cycle vs. 1197mg/cycle), and the highest treatment costs per cycle ($14003.70/cycle for the US, $52736.25/cycle for China) (UpToDate, 2023). The costs of adebrelimab per cycle in China was significantly lower than that of other drugs in the table. From the US perspective, in order to make adebrelimab’s costs per cycle lower than durvalumab, the Xmax needed to be reduced to 7001.85, at which adebrelimab plus chemotherapy remained an absolutely economic option.

### 3.5 Subgroup analysis

We analyzed the economics of adebrelimab plus chemotherapy in different subgroups from the perspectives of healthcare sector in the US and Chinese basing on the current market price (only had the market price of adebrelimab in China) and estimated Xmax of adebrelimab (Table 3). From the Chinese perspective, at the adebrelimab price of $1382.82, the economics of adebrelimab plus chemotherapy in each subgroup was consistent with the overall population, and no statistical differences were found among subgroups. However, basing on the estimated Xmax at the WTP threshold of $37,422, subgroup analysis found significant differences in patients with different ages, smoking histories, and status of liver metastases (*p* < 0.05). From the US perspective, model 1 showed significant differences among subgroups with different ages, sex, ECOG performance status, smoking histories, LDH concentration at enrollment, status of liver metastases, and PD-L1 tumor proportion scores (*p* < 0.05). In model 2, only significant differences were found in patients with different status of liver metastases (*p* < 0.001).

**TABLE 3:** Results of subgroup analysis.

Analysis Perspective	HR for OS (95% CI)	HR for PFS (95% CI)	China								United States			
			Model 1^a^		Model 2^a^		Model 1^b^		Model 2^b^		Model 1^b^		Model 2^b^	
			ICER, $/QALY (range)	Cost-effectiveness probability of adebrelimab, %	ICER, $/QALY (range)	Cost-effectiveness probability of adebrelimab, %	ICER, $/QALY (range)	Cost-effectiveness probability of adebrelimab, %	ICER, $/QALY (range)	Cost-effectiveness probability of adebrelimab, %	ICER, $/QALY (range)	Cost-effectiveness probability of adebrelimab, %	ICER, $/QALY (range)	Cost-effectiveness probability of adebrelimab, %
**Age**				*p*-value = 1.00		*p*-value = 1.00		*p*-value = 0.02		*p*-value = 0.02		*p*-value< 0.001		*p*-value = 1.00
<65 years	0.71 (0.54.0.93)	0.70 (0.54,0.91)	114488.30 (49332.50,dominated)	0.10%	230743.97 (97490.80,dominated)	0.00%	96342.82 (41813.80, dominated)	0.70%	95562.06 (41484.60, dominated)	0.70%	64068.00 (3158.83, 93764.90)	40.50%	1220182.88 (189491.00, dominated)	0.00%
≥65 years	0.70 (0.48,1.00)	0.62 (0.43,0.89)	144397.20 (45898.30,dominated)	0.00%	283024.75 (88861.30,dominated)	0.00%	122762.00 (39190.90, dominated)	0.00%	121837.37 (38897.60, dominated)	0.00%	39204.90 (predominant,49093.61)	79.40%	1510637.43 (102132.00, dominated)	0.00%
**Sex**				*p*-value = 0.37		*p*-value = 1.00		*p*-value = 0.29		*p*-value = 0.34		*p*-value< 0.001		*p*-value = 1.00
Male	0.72 (0.57–0.92)	0.72 (0.57,0.90)	104510.30 (50328.60,dominated)	0.10%	211236.26 (99542.10,dominated)	0.00%	87851.81 (42645.30, dominated)	0.60%	87133.83 (42309.10, dominated)	0.70%	75294.09 (predominant,110274.00)	30.80%	1081565.78 (205669.00, dominated)	0.00%
Female	0.62 (0.37,1.05)	0.55 (0.33,0.90)	1130910.00 (43517.40,dominated)	0.40%	2218498.39 (83528.70,dominated)	0.00%	961189.10 (37270.70, dominated)	0.20%	953978.79 (36997.40, dominated)	0.30%	96924.76 (18250.30, 183247.00)	93.00%	13560570.20 (54709.20, dominated)	0.00%
**ECOG performance status**				*p*-value = 1.00		*p*-value = 1.00		*p*-value = 0.01		*p*-value = 0.12		*p*-value< 0.001		*p*-value = 1.00
0	0.83 (0.46,1.52)	0.62 (0.35,1.10)	79562.05 (32413.30,dominated)	0.00%	149365.46 (59963.60,dominated)	0.00%	68668.06 (28110.90, dominated)	0.00%	68202.47 (27919.60, dominated)	0.00%	100028.00 (predominant,190549.40)	100.00%	470017.64 (predominant, dominated)	0.00%
1	0.69 (0.55,0.87)	0.69 (0.56,0.87)	134114.10 (55676.10,dominated)	0.10%	271679.39 (111048.00,dominated)	0.00%	112642.70 (47031.70, dominated)	0.80%	111719.67 (46654.40, dominated)	0.40%	83154.84 (72877.60, 98531.60)	4.80%	1542692.72 (302435.00, dominated)	0.00%
**Smoking history**				*p*-value = 0.50		*p*-value = 1.00		*p*-value< 0.001		*p*-value = 0.03		*p*-value = 0.02		*p*-value = 1.00
Current or former smoker	0.75 (0.59,0.95)	0.76 (0.60,0.96)	85728.21 (46785.10,dominated)	0.20%	173389.38 (92759.60,dominated)	0.00%	72044.74 (39606.90, dominated)	1.80%	71453.00 (39291.30, dominated)	0.60%	31541.30 (predominant,139191.00)	51.90%	796984.23 (158540.00, dominated)	0.00%
Never smoked	0.59 (0.37,0.95)	0.44 (0.27,0.71)	dominated (56043.60,dominated)	0.00%	dominated (105263.00,dominated)	0.00%	dominated (48361.20, dominated)	0.00%	dominated (48030.40, dominated)	0.00%	40110.90 (predominant,83514.90)	46.40%	dominated (176196, dominated)	0.00%
**LDH concentration at enrolment**				*p*-value = 1.00		*p*-value = 1.00		*p*-value = 1.00		*p*-value = 1.00		*p*-value< 0.001		*p*-value = 1.00
≤ULN	0.59 (0.42,0.82)	0.70 (0.52,0.95)	3368594.00 (58604.30,dominated)	0.00%	7239725.89 (120749.00,dominated)	0.00%	2764377.00 (48901.50, dominated)	0.00%	2738378.84 (48475.20, dominated)	0.00%	40343.10 (predominant,104485.30)	0.00%	39453957.30 (424167.00, dominated)	0.00%
>ULN	0.83 (0.62,1.11)	0.64 (0.48,0.85)	77168.73 (42680.90,dominated)	0.00%	145955.09 (80319.70,dominated)	0.00%	66433.19 (36805.00, dominated)	0.00%	65973.60 (36549.00, dominated)	0.00%	18045.20 (predominant,30180.65)	97.50%	461862.26 (10992.90, dominated)	0.00%
**Liver metastases**				*p*-value = 1.00		*p*-value = 1.00		*p*-value< 0.001		*p*-value< 0.001		*p*-value< 0.001		*p*-value< 0.001
Yes	0.92 (0.65,1.31)	0.74 (0.51,1.07)	56901.71 (34890.10,451566.00)	0.10%	108515.89 (65772.20,855030.00)	0.00%	48845.20 (30067.60, 388607.00)	2.80%	48497.38 (29853.70, 385942.00)	2.80%	45461.16 (555.62, 61992.90)	100.00%	220141.70 (predominant,5039119.00)	5.70%
No	0.61 (0.46,0.81)	0.64 (0.50,0.83)	754648.00 (65521.20,dominated)	0.00%	1558544.84 (132075.00,dominated)	0.00%	629183.10 (55131.50, dominated)	0.00%	623811.91 (54679.50, dominated)	0.10%	25042.50 (predominant,78162.40)	0.00%	10695836.90 (477105.00, dominated)	0.10%
**Brain metastases**														
No	0.68 (0.55,0.85)	0.65 (0.53,0.81)	159532.10 (60674.90,dominated)	0.10%	319445.66 (120109.00,dominated)	0.00%	134573.90 (51396.90, dominated)	0.00%	133504.56 (50994.00, dominated)	0.10%	27360.56 (predominant,104422.00)	1.40%	1853794.24 (358557.00, dominated)	0.00%
**Disease stage**														
IV	0.72 (0.58,0.90)	0.68 (0.55,0.83)	111606.50 (54415.10,dominated)	0.00%	222336.93 (106664.00,dominated)	0.00%	94323.77 (46258.50, dominated)	0.20%	93581.40 (45903.70, dominated)	0.10%	9229.36 (predominant,85020.90)	17.70%	1124819.92 (245840.00, dominated)	0.00%
**PD-L1 tumour proportion score**				*p*-value = 1.00		*p*-value = 1.00		*p*-value = 1.00		*p*-value = 0.75		*p*-value< 0.001		*p*-value = 1.00
<1%	0.66 (0.52,0.83)	0.68 (0.54,0.85)	183382.00 (61363.10,dominated)	0.10%	375342.14 (123427.00,dominated)	0.00%	153421.10 (51674.20, dominated)	0.30%	152134.11 (51252.00, dominated)	0.60%	19070.80 (predominant,45291.21)	0.00%	2361549.59 (408089.00, dominated)	0.00%
≥1%	0.72 (0.33,1.59)	0.70 (0.34,1.45)	107880.80 (29987.60,dominated)	0.10%	216508.91 (56315.10,dominated)	0.00%	90925.80 (25874.40, dominated)	0.20%	90196.27 (25686.30, dominated)	0.40%	64967.80 (predominant,93277.80)	100.00%	1102113.68 (predominant, dominated)	0.00%

^a^ adebrelimab price was $1382.82.

^b^ adebrelimab price was the estimated X_max_ at the WTP threshold of $37,422 for China and $100,000 for the US.

## 4 Discussions

As the results of phase-III clinical trials such as IMpower133 and CASPIAN were reported, increasing attention was attracted by the positive activity of ICIs in the development of immunogenic tumor clones and elicitation of adaptive immune responses for ES-SCLC (Paz-Ares et al., 2019; National Institute for Health and Care Excellence, 2020). Therefore, atezolizumab and durvalumab have been approved and quickly became the dominant treatment options in patients with ES-SCLC. Inspiring results of the CAPSTONE-1 trial make adebrelimab, a domestically produced ICIs, being successfully approved and become the fifth PD-L1 monoclonal antibody with approval in China (Wang et al., 2022). Although most patients discontinued adebrelimab plus chemotherapy (93%) or chemotherapy alone (99%) as post-study treatment in the trial, the clinical benefits of OS and PFS were sustained and supported the cost-effectiveness of adebrelimab combination regimen. Considering the potential gap between the costs of adebrelimab plus chemotherapy and chemotherapy, we performed the study to evaluate the cost-effectiveness of adebrelimab plus chemotherapy vs. chemotherapy alone as the first-line therapy for ES-SCLC by simulating the long-term survival with partitioned models basing on the CAPSTONE-1 trial. We referred to the research conducted by Shao et al., which found that serplulimab plus chemotherapy were probably only cost-effective for ES-SCLC in China, to construct the economic evaluation model (Shao et al., 2023). This model optimized the fitting and extrapolation of survival curves, reducing the uncertainty associated with treatment utility due to missing QoL data. It proposed a feasible approach for conducting economic evaluations of drugs with unknown prices and generated quantitative evidence for pricing decisions regarding new drugs.

Currently, there is an economic article, however, whose results differ to this study (You et al., 2022). This is mainly because the methods of economic evaluation are different between two studies. Firstly, You et al. only considered four fitting models, namely, exponential, Weibull, log-normal, and log-logistic, and the log-logistic model was the best one based on the principle of lower AIC and BIC values. This study fitted 10 models and comprehensively evaluated the performance based on AIC, log likelihood, and MSE. Ultimately, the flexible and data distribution assumption-free RP model was selected, which exhibited significantly better fitting and extrapolation performance compared to the log-logistic model. Secondly, the cost of adebrelimab in this study was based on known market prices, which yielded results that are closer to reality. However, You et al. assumed adebrelimab price was artificially to be 0.73 times the market price. Furthermore, this study also predicted the economically viable adebrelimab prices under different WTP thresholds, providing a basis for pricing decisions for the medication. Thirdly, due to the discrepancy between the median duration of drug use and the planned maximum duration, we separately considered the scenarios of 8 cycles or 2 years. This aspect was not addressed in the research conducted by You et al. Fourthly, since patients’ QoL tends to decrease as time to death shortens, we simultaneously considered effectiveness based on both PB-utility and TTD-utility. Sensitivity analysis also suggested the importance of evaluating the economics of regimens from the perspective of TTD-utility. In contrast, You et al. focused only on PB-utility. Fifthly, certain monoclonal antibody drugs have been included in PAP in China, such as Pembrolizumab, Trastuzumab, Rituximab, *etc.* Compared to other similar drugs, adebrelimab, an innovative drug developed in China, has comparable efficacy, lower cost. There is a chance for it to be included in the Chinese PAP. Therefore, this study also evaluated the economics of regimens in the scenario of Chinese PAP. Currently, adebrelimab is not yet available in the US and is still undergoing clinical research. It is unknown whether it will have the opportunity to be included in the US PAP. Hence, this study did not consider the scenario of the US PAP. You et al. did not focus on the impact of PAP on the economics of regimens. Sixthly, this study selected second-line treatment with a reported frequency of >10% in the CAPSTONE-1 trial and considered the actual treatment costs corresponding to effectiveness. Sensitivity analysis also demonstrated that the costs of second-line treatment was an important factor affecting the results. In contrast, You et al. only considered irinotecan + cisplatin or BSC, neglecting the costs of other second-line treatment with higher frequencies of use. Seventhly, this study considered the impact of various adebrelimab price on the results in subgroup analyses, aiming to provide additional insights for drug pricing decisions. You et al. assumed only one possible price for adebrelimab without further testing or estimation.

The current market price of adebrelimab in China did not support the conclusion that adebrelimab plus chemotherapy was an economic option comparing with chemotherapy at the WTP threshold of $37,422/AQLY. This conclusion was not affected by utility-related methods and patients characteristics, but it could be changed by the implementation of PAP. Although the costs of adebrelimab per cycle in China was significantly lower than that of other similar drugs already on the market, it is still necessary to reduce adebrelimab market price in order to make adebrelimab plus chemotherapy an economic treatment option comparing with chemotherapy out of PAP. Since adebrelimab price in the US is unknown, the study did not include adebrelimab price in the OWSA. However, by observing the relationship between adebrelimab price and ICER, it could be found that the higher the adebrelimab price, the greater the ICER Supplementary Figure S3). Therefore, from the US perspective, in order to achieve the economics of adebrelimab plus chemotherapy at the WTP threshold of $100,000/QALY, it is necessary to determine the upper limit of adebrelimab price. When it is assumed that adebrelimab price in the US was $1382.82/600mg, adebrelimab plus chemotherapy had an absolute economic advantage comparing with chemotherapy. However, this price was significantly lower than the estimated Xmax at the WTP threshold of $100,000 for the US, causing increased costs of adebrelimab plus chemotherapy comparing with chemotherapy ($17394.10/600 mg of model 1 and $8894.98/600 mg of model 2 in base-case, $17393.80/600 mg of model 1 and $8912.51/600 mg of model 2 in scenario 1), which was higher than the Xmax calculated basing on the costs of durvalumab per cycle ($7001.85/600 mg).

The CASPIAN comparing with the CAPSTONE-1 found that durvalumab plus chemotherapy (etoposide plus carboplatin or cisplatin) vs. chemotherapy as the first-line therapy for ES-SCLC showed a similar mortality risk reduction of OS (0.71 of HR in the CASPIAN vs. 0.72 of HR in the CAPSTONE-1), and a lower mOS (12.9 months in the CASPIAN with durvalumab plus chemotherapy vs. 15.3 months in the CAPSTONE-1 with adebrelimab plus chemotherapy, 10.5 months in the CASPIAN with chemotherapy vs. 12.8 months in the CAPSTONE-1 with chemotherapy) (Paz-Ares et al., 2022; Wang et al., 2022). From the US healthcare sector perspective, two cost-effectiveness analyses basing on the CASPIAN considered the cost of durvalumab, the utilities of PFS and PD, and the discount rate as the main factors that could affect the results, but they could not change the economic conclusion of durvalumab plus chemotherapy comparing with chemotherapy at the WTP threshold of $100,000/QALY. In the OWSA of this study, the utilities of PFS and PD, and the discount rate were also the main factors that could affect the ICERs of adebrelimab plus chemotherapy comparing with chemotherapy, but could not change its economic conclusion.

The base-case analysis of this study showed that the total costs of adebrelimab plus chemotherapy and chemotherapy mainly came from the PD and death states. The results of OWSA found that the cost of palliative care was the primary factor that could affect the results of cost-effectiveness analysis, and its impact was significantly higher than other factors, even could reverse the economic conclusion of adebrelimab plus chemotherapy comparing with chemotherapy. The cost of palliative care was the primary component of the total costs of both strategies, and the death-related treatment costs (total costs of palliative care) were significantly higher in chemotherapy group than in adebrelimab group, which were related to the higher mortalities in chemotherapy group. The OS and PFS of patients receiving durvalumab plus chemotherapy in the CASPIAN were not significantly different from those receiving chemotherapy in the CAPSTONE-1 during the 2-year follow-up period, which indicated that the death-related treatment costs of these two groups were similar. If it is assumed that the treatment regimens received by patients during the PD state in the CASPIAN was the same as that in the CAPSTONE-1 (CASPIAN did not report specific subsequent treatment regimens), their PD-related costs would be similar.

In the base-case analysis, the incremental QALYs of adebrelimab plus chemotherapy comparing with chemotherapy mainly came from the PFS state. By comparing the PFS of patients with adebrelimab plus chemotherapy and of patients with durvalumab plus chemotherapy during the same follow-up period, it was found that the PFS of patients with adebrelimab plus chemotherapy was significantly higher than that of patients with durvalumab plus chemotherapy (*p* = 0.04, Supplementary Figure S4). Therefore, under the same utility of PFS, adebrelimab plus chemotherapy could produce higher PFS-related QALYs. If the utility of PD was the same, the total QALYs produced by adebrelimab plus chemotherapy would be higher than that of durvalumab plus chemotherapy. At the same WTP threshold, it is acceptable for adebrelimab plus chemotherapy to generate higher PFS-related costs than durvalumab plus chemotherapy. Therefore, the upper limit of adebrelimab price, higher than $7001.85/600mg, estimated in this study was reasonable.

Subgroup analysis found that from the US healthcare sector perspective, the cost-effectiveness models basing on the median cycle number of drugs delivered in the CAPSTONE-1 were sensitive to age, sex, ECOG performance status, smoking history, LDH concentration at enrolment, status of liver metastases, and PD-L1 tumor proportion score. The results were related to the differences of OS and PFS among subgroups. Lower HR of OS meant greater differences of mortalities and death-related treatment costs between adebrelimab plus chemotherapy and chemotherapy, and higher HR of PFS meant greater incremental QALYs of adebrelimab plus chemotherapy comparing with chemotherapy. Therefore, there were significant differences in the cost-effectiveness probability of adebrelimab plus chemotherapy among these subgroups. However, not all models were sensitive to these characteristics. From the Chinese healthcare sector perspective, the results of models basing on adebrelimab market price were not sensitive to these characteristics, but the results of models basing on adebrelimab estimated prices had significant differences in patients with different ages, smoking histories, and status of liver metastases. This indicated that adebrelimab price was also one of the factors that could affect the economics of adebrelimab plus chemotherapy comparing with chemotherapy. It is worth noting that when adebrelimab reaches Xmax, the probability of adebrelimab plus chemotherapy becoming a cost-effective treatment option in the liver metastasis population was significantly higher than in the non-liver metastasis population. This is mainly due to the higher incremental QALYs of 0.29 produced by adebrelimab plus chemotherapy, which was 15.58 times higher in the liver metastasis population compared to the non-liver metastasis population and significantly higher than all other subgroups. Liver metastasis has a relatively high incidence in ES-SCLC patients, approximately 43.10% (Valette et al., 2023). Currently, there is limited research on the improvement of liver metastasis prognosis in ES-SCLC patients with PD-1/PD-L1 inhibitors. Apart from adebrelimab plus chemotherapy, atezolizumab plus chemotherapy has also shown positive effects (no liver metastases at baseline vs. liver metastases, OR = 0.59, *p* = 0.069), suggesting that PD-1/PD-L1 plus chemotherapy could be a potential treatment option for ES-SCLC patients with liver metastasis (Reck et al., 2022). Further evidence is needed to better confirm this point.

## 5 Limitations

This study has several limitations. First, the CAPSTONE-1 only included the Chinese (Wang et al., 2022), and it is not known whether the survival data obtained from this population is consistent with that of the American, thus, the survival benefits estimated from the US healthcare sector perspective in this study may have some biases. Second, only the regimens used by a large number of patients were considered in the subsequent treatment. This may lead to biases in the estimation of subsequent treatment-related costs. Third, the utilities used in the models came from published literature rather than the CAPSTONE-1 (Nafees et al., 2017; National Institute for Health and Care Excellence, 2020; Vedadi et al., 2021), and whether they were applicable to patients in the CAPSTONE-1 was not known. Additionally, the study assumed that there was no difference in the utilities between patients receiving adebrelimab plus chemotherapy and chemotherapy, but the CAPSTONE-1 found significant differences in OS and PFS between two groups (Wang et al., 2022). This assumption may underestimate the incremental QALYs. Fourth, the study only considered some serious TRAEs, and it assumed that the other adverse events (AEs) had a very small impact on the results (Su et al., 2021; Shao et al., 2023). However, the overall incidence of AEs (including TRAEs and immune-related AEs) in patients with adebrelimab plus chemotherapy is higher than that in patients with chemotherapy (Wang et al., 2022). This assumption may overestimate the ICERs of adebrelimab plus chemotherapy comparing with chemotherapy. Since the CAPSTONE-1 did not report the treatment regimens for severe TRAEs, their costs were unknown. Therefore, this study referred to previous literature to estimate them, which may lead to some biases in the calculation of TRAEs-related costs (Shao et al., 2023). However, the results of OWSA suggested that these biases had a relatively limited impact on the results. Fifth, the economic evaluation of adebrelimab plus chemotherapy in this study was based only on the CAPSTONE-1, and the estimated adebrelimab price was only based on the cost-effectiveness models (Wang et al., 2022; Shao et al., 2023). The availability and affordability of this treatment regimen in the US and China for ES-SCLC still need to be demonstrated through additional studies.

## 6 Conclusion

From the Chinese healthcare sector perspective, if PAP was not taken into account, the current adebrelimab market price cannot make adebrelimab plus chemotherapy cost-effective comparing with chemotherapy, unless adebrelimab price was reduced by at least $202.03/600 mg when health benefits were calculated by PB-utility or $103.06/600 mg when health benefits were calculated by TTD-utility. If PAP was considered, adebrelimab plus chemotherapy was cost-effective comparing with chemotherapy. From the US healthcare sector perspective, if adebrelimab price was lower than $18592.00/600 mg when health benefits were calculated by PB-utility or $20832.10/600 mg when health benefits were calculated by TTD-utility, adebrelimab plus chemotherapy may become a cost-effective treatment option comparing with chemotherapy. The incremental QALYs calculated from the Chinese perspective was similar to that calculated from the US perspective, but there was a significant difference in incremental costs. This is primarily due to the higher cost of palliative care in the US. This cost was associated with mortality rates and palliative care per patient. The cost of palliative care per patient in the US was 14.79 times higher than in China, and the mortality rate for chemotherapy was significantly higher than that for adebrelimab plus chemotherapy. Therefore, the estimated upper limit of adebrelimab price from the US perspective was significantly higher than in China. This study provided reference for the pricing decision of adebrelimab and the development of clinical treatment strategies for ES-SCLC. However, this study still has some limitations. More long-term clinical studies are needed to verify and improve the results, and the estimation of adebrelimab price needs to be considered in conjunction with more evidence that may affect the availability and affordability of it.

## Figures and Tables

**Table:** 

**SCLC**	Small-cell lung cancer
**ES-SCLC**	Extensive-stage small-cell lung cancer
**WTP**	Willingness-to-pay
**QALYs**	Quality-adjusted life years
**PB-utility**	Progression-based utility
**TTD-utility**	Time-to-death utility
**OS**	Overall survival
**mOS**	Median OS
**ICIs**	Immune checkpoint inhibitors
**CTLA-4**	Cytotoxic T-lymphocyte antigen 4
**PD-1/PD-L1**	Programmed cell death-1/programmed cell death receptor ligand-1
**TMB**	Tumor mutational burden
**NCCN**	National Comprehensive Cancer Network
**CSCO**	Chinese Society of Clinical Oncology
**CDE**	Centre for Drug Evaluation
**PD**	Progressive disease
**PFS**	Progression-free survival
**ICERs**	Incremental cost-effectiveness ratios
**IPD**	Individual patient data
**FP**	Fractional polynomial
**RCS**	Restricted cubic spline
**RP**	Royston-parmar spline
**AIC**	Akaike information criterion
**MSE**	Mean squared errors
**TRAEs**	Treatment-related adverse events
**CMS**	Centers for Medicare & Medicaid Services
**AWP**	Average Wholesale Price
**QoL**	Quality of life
**EQ-5D**	EuroQoL-5D
**OWSA**	One-way sensitivity analysis
**ISPOR-SMDM**	International Society for Pharmacoeconomics and Outcomes Research-Society for Medical Decision Making
**PAP**	Patient assistance program
**ECOG**	Eastern Cooperative Oncology Group
**LDH**	Lactate dehydrogenase
**BSC**	Best supportive care

## Data Availability

The datasets presented in this study can be found in online repositories. The names of the repository/repositories and accession number(s) can be found in the article/Supplementary material.
