# Supplementary material for: Adebrelimab plus chemotherapy vs. chemotherapy for treatment of extensive-stage small-cell lung cancer from the US and Chinese healthcare sector perspectives: a cost-effectiveness analysis to inform drug pricing
Source: Front Pharmacol. 2023 Jul 20;14:1241130. doi: 10.3389/fphar.2023.1241130 (PMC10398383; doi:10.3389/fphar.2023.1241130)
Supplement: Supplementary file 1 [file DataSheet1.pdf]

Supplementary Material

Adebrelimab Plus Chemotherapy vs Chemotherapy for Treatment of Extensive-Stage Small-Cell Lung Cancer from the US and Chinese Healthcare Sector Perspectives: A Cost-Effectiveness Analysis to Inform Drug Pricing

Yena Gan[1], Fenghao Shi[2,3], He Zhu[2,3], Sheng Han[2,3], Duoduo Li[1*]

[1]Dongzhimen Hospital, Beijing University of Chinese Medicine, Beijing, China

[2]International Research Center for Medicinal Administration, Peking University, Beijing, China

[3]School of Pharmaceutical Sciences, Peking University, Beijing, China

*** Correspondence:**
Duoduo Li
tarako@163.com

Methods

1. Treatment regimens in the CAPSTONE-1 trial

In the CAPSTONE-1 trial, a total of 462 patients were stratified by liver metastases, brain metastases, and lactate dehydrogenase (LDH) concentration at environment and were randomly assigned (1:1) to receive adebrelimab plus chemotherapy (230 patients) or chemotherapy (232 patients). Per the CAPSTONE-1 protocol, patients were treated with adebrelimab or placebo (20 mg/kg, day 1) combining with carboplatin (area under the curve [AUC] 5 mg/mL per min, day 1) and etoposide (100 mg/m² per day, day 1–3) every 3 weeks for 4-6 cycles, followed by maintenance therapy with adebrelimab or placebo (20 mg/kg, day 1) per cycle.

2. Demographics of patients

Basic clinical information was collected from the CAPSTONE-1 trial (**eTable 1**). The following demographics of patients would be matched to a hypothetical patient cohort for our subsequent analysis: 62 years old (median age), histologically or

cytologically confirmed ES-SCLC, without previously systemic treatment, with an Eastern Cooperative Oncology Group (ECOG) performance status score of 0 or 1, assumed area under the concentration curve of 5 mg/mL/min, serum creatinine of 1, weight 65 kg for Chinese and 70 kg for American, and body surface area (BSA) 1.72 m^2 for Chinese and 1.79 m^2 for American.

3. Costs of treatment

3.1 Costs of subsequent treatment regimens

The CAPSTONE-1 reported about 30 subsequent treatment regimens, but most regimens were used by few patients, and some price information for drugs cannot be accessed through public databases. Additionally, the CAPSTONE-1 trial was conducted in China, and some drugs used in the study had not yet been approved for sale in the US, resulting in missing price information. To reduce the uncertainty in the estimation of adebrelimab price due to this portion of uncertain information, we only included drugs with a usage percentage of 10% or greater in the models. The dosage and frequency of each drug were based on the drug monograph (**eTable 2**).

The drugs usage percentages included their usage percentages in monotherapy and combination therapies. The usage percentages of other drugs in combination therapy were relatively low and had not been included. We assumed that the costs of treating progressive disease (PD) were the total costs of using each of the above-mentioned drugs for subsequent treatment, and the cost of the drug was equal to the cost of the drug multiplied by the patient percentage who used it. Only 67.53% of patients in the study reported subsequent treatment, and the specific treatment regimens for the other patients were unknown. We assumed that these patients received best supportive care (BSC) (38.70% in adebrelimab group and 26.29% in chemotherapy group). Since the CAPSTONE-1 did not report the duration of subsequent treatment, we assumed that patients received the above-mentioned drugs from PD until 1 month before death.

3.2 Cost of each drug per cycle

The cost of the drug per cycle was equal to the product of the unit packaging cost of the drug and the minimum package number. The minimum package number should be the smallest positive integer that contains the average dosage of the drug. For carboplatin, for example, the average dosage used by patients with adebrelimab plus chemotherapy was 485mg per cycle, and the minimum package number required was 10 ($9 < \frac{485}{50} < 10$). Therefore, the cost of carboplatin for this portion of patients each cycle was \$26.7 (\$2.67 × 10 = \$26.7). The costs of placebo-related drug acquisition and administration were not included.

3.3 Treatment duration of drugs

Changes in the duration of regimens with different costs may affect the calculation of total treatment-related costs. According to the protocol, patients could continue to receive adebrelimab or placebo for up to 2 years, unless disease progression or intolerable drug toxicity was observed. However, the median cycle number of adebrelimab or placebo reported in the trial was 8 cycles. To reduce the impact of uncertain assumptions about the treatment duration on results, we simultaneously considered the treatment duration for 2 years and 8 cycles. The following assumptions were made in the base-case analysis: (1) model 1: patients recovered after receiving 8 cycles of adebrelimab or placebo, or discontinued treatment due to intolerable drug toxicity, or disease progression, or death; (2) model 2: patients recovered after receiving adebrelimab or placebo treatment for 2 years, or discontinued treatment due to intolerable drug toxicity, or disease progression, or death.

3.4 Costs of follow up

The costs of follow-up mainly consisted of two aspects: laboratory tests and imaging exams. Laboratory tests, including electrocardiograms, haematological examination, liver and renal function, coagulation, and thyroid function tests, were performed every 6 weeks. Imaging exams, including CT or MRI, were performed every 6 weeks for the first year, and then every 9 weeks after that. We assumed that all patients were followed up at the above-mentioned frequency until disease progression or discontinued treatment. After the first year, the follow-up frequency was changed to every 9 weeks for the next 2 years, every 3 months for the next 3 years, every 6 months for the next 3 years, and then annually after that.

4. Methodology of external validation and the final selection of extrapolation models

The survival results of long-term follow-up were not provided in the CAPSTONE-1, which would be obtained from the fitted models. To ensure the credibility of models fitting results, we considered selecting models by two steps. Firstly, the best survival model was selected based on the minimum akaike information criterion (AIC) and maximum Log likelihood. Then, external data were used to verify the clinical rationality of models' extrapolation results and to calibrate the models' selection. In this case, mean squared errors (MSE) were chosen as the evaluation index, the smaller the MSE value, the better the model performance. CASPIAN had the longest follow-up period in published clinical studies related to immunotherapy (27 months of PFS and 42 months of OS), and it was selected as an external data source. We evaluated the rationality of PFS extrapolation results within

the range of 6 to 27 months, and the OS extrapolation results within the range of 15 to 42 months.

For the OS data of patients with adebrelimab plus chemotherapy, we selected "RP(royston-parmar spline)-hazard-1" as the survival model, which had no significant difference in fitting and extrapolation performance comparing with the best model (RCS1), but had significant advantages in the clinical rationality of extrapolation results. The other three groups selected the survival models with the best fitting and extrapolation performance, and the extrapolation results generated by them all had good clinical rationality.

eTable 1 Baseline characteristics of patients in the CAPSTONE-1 trial

	**Adebrelimab group (n=230)**	**Chemotherapy group (n=232)**
**Age, number (proportion)**		
<65	155 (67.39)	147 (63.36)
≥65	75 (32.61)	85 (36.64)
**Sex, number (%)**		
Male	184 (80.00)	188 (81.03)
Female	46 (20.00)	44 (18.97)
**ECOG performance status, number (%)**		
0	33 (14.35)	30 (12.93)
1	197 (85.65)	202 (87.07)
**Smoking history, number(%)**		
Former and current smoker	180 (78.26)	179 (77.16)
Never smoked	50 (21.74)	53 (22.84)

**LDH concentration at enrolment, number (%)**		
>ULN	114 (49.57)	117 (50.43)
≤ULN	116 (50.43)	115 (49.57)
**Liver metastases, number (%)**		
Yes	73 (31.74)	74 (31.90)
No	157 (68.26)	158 (68.10)
**PD-L1 tumor proportion score, number (%)**		
<1%	196 (85.22)	200 (86.21)
≥1%	24 (10.43)	20 (8.62)
**Brain metastases, number (%)**		
No	225 (97.83)	227 (97.84)
**Disease stage, number (%)**		
IV	222 (96.52)	226 (97.41)
**Treatment duration, median cycles**		
Adebrelimab or placebo	8 (6,12)	8 (6,10)
Etoposide	6 (4,6)	6 (4,6)
Carboplatin	6 (4,6)	6 (4,6)
**Delivered dose intensity, (mean ± SD), mg/3-week**		
Adebrelimab or placebo	1197±229	1147±251

Etoposide	445±76	438±79
Carboplatin	489±122	485±119
**Relative dose intensity, (mean±SD), %**		
Adebrelimab or placebo	100.0±0.3	100.0±0.5
Etoposide	99.9±0.6	99.8±0.8
Carboplatin	99.9±0.4	99.9±0.5

ECOG, Eastern Cooperative Oncology Group; LDH, lactate dehydrogenase; PD-L1, programmed cell death receptor ligand-1; SD, standard deviation.

eTable 2 Usage and proportion of drugs for subsequent treatment

**Drug**	**Usage**	**Adebrelimab plus chemotherapy (%)**	**Chemotherapy (%)**
Irinotecan	350 mg/m^2, Q3W	25.65	37.50
Cisplatin	75 mg/m^2, Q3W	14.35	17.67
Carboplatin	AUC 5 mg/mL/min, Q3W	\	10.34
Etoposide	100mg/m^2, d1-3, Q3W	\	10.78

Q3W, every 3 weeks; AUC, area under curve; d, day

eTable 3 Goodness-of-fit, extrapolation performance and clinical rationality results of parametric survival models

	**OS**						**PFS**					
**Model**	**Adebrelimab group**			**Chemotherapy group**			**Adebrelimab group**			**Chemotherapy group**		
	**AIC**	**Log likelihood**	**MSE**	**AIC**	**Log likelihood**	**MSE**	**AIC**	**Log likelihood**	**MSE**	**AIC**	**Log likelihood**	**MSE**

**Exponential**	173.03	-85.52	215.89	261.44	-129.72	76.54	254.51	-126.25	80.62	293.96	-145.98	51.29
**Weibull**	138.07	-67.04	74.00	191.02	-93.51	37.07	246.46	-121.23	76.51	215.92	-105.96	11.19
**Gamma**	136.86	-66.43	**51.01**	189.45	-92.73	26.67	236.23	-116.11	66.47	198.88	-97.44	7.48
**Log-normal**	147.36	-71.68	85.97	217.95	-106.98	30.83	210.26	-103.13	36.63	217.76	-106.88	7.03
**Gompertz**	153.08	-74.54	142.77	215.51	-105.75	54.43	256.01	-126.01	80.19	271.37	-133.68	29.02
**Log-logistic**	133.85	-64.92	**36.25**	182.56	-89.28	17.21	199.51	-97.76	23.13	174.68	-85.34	2.91
**Generalized gamma**	139.27	-66.63	**46.25**	190.99	-92.49	27.85	213.20	-103.60	34.19	200.88	-97.44	6.73
**FP1-1**	174.67	-85.33	179.85	254.10	-125.05	62.78	244.50	-120.25	79.39	223.23	-109.62	13.16
**FP1-2**	140.37	-68.18	56.77	199.27	-97.63	29.09	237.70	-116.85	70.41	238.31	-117.16	14.64
**FP2-1**	**123.69**	**-58.84**	516.22	182.88	-88.44	23.23	187.47	-89.73	18.58	176.55	-85.28	**0.37**
**FP2-2**	**123.91**	-58.96	469.63	178.03	-86.02	28.40	184.35	-89.17	19.05	184.61	-89.31	1.06
**RCS1**	**123.67**	**-58.83**	400.30	172.77	-83.39	46.40	194.39	-94.20	15.31	167.12	-80.56	**0.33**
**RCS2**	125.54	**-58.77**	440.89	171.70	-81.85	27.22	181.39	-86.70	23.29	165.83	-78.91	**0.19**
**RP-hazard-1**	128.39	-60.19	103.53	**171.61**	-81.80	**14.46**	**158.68**	**-72.34**	**8.07**	148.29	**-67.15**	1.32
**RP-hazard-2**	130.12	-60.06	133.70	**171.62**	**-80.81**	**13.51**	160.35	-74.17	8.57	**146.85**	**-67.43**	1.37
**RP-odds-1**	128.61	-60.31	107.41	**170.44**	-81.22	**14.78**	**157.37**	**-71.68**	**7.23**	147.57	**-66.78**	1.64
**RP-odds-2**	130.06	-60.03	142.94	172.05	**-81.02**	14.93	**159.26**	-73.63	8.23	**146.89**	-67.44	1.43
**RP-normal-1**	130.67	-60.34	141.72	173.54	**-79.77**	18.09	159.65	**-72.82**	**7.68**	147.67	-66.84	1.48
**RP-normal-2**	130.59	-61.29	90.76	217.95	-106.98	30.83	159.29	-73.64	8.25	**147.02**	-67.51	1.40

OS, overall survival; PFS, progression-free survival; AIC, akaike's information criterion; MSE, mean squared errors; FP, fractional polynomial; RCS, restricted cubic spline; RP, royston-parmar spline.

eTable 4 Baseline values, ranges, and distributions of model parameters

Parameters	Baseline value	Low	Upper	Distribution	Source

**Costs from US healthcare sector perspective**					
**Costs of drugs, $/cycle**					
Adebrelimab (100 mg)	X	X_{min}	X_{max}	gamma	
Irinotecan (20 mg)	2.60	2.08	3.12	gamma	[1]
Cisplatin (10 mg)	1.92	1.53	2.30	gamma	[1]
Carboplatin (50 mg)	2.67	2.13	3.20	gamma	[1]
Etoposide (10 mg)	0.84	0.67	1.01	gamma	[1]
**Costs of administration**					
Laboratory/time	111.65	65.42	185.44	gamma	[1]
Imaging/time	438.21	207.90	709.23	gamma	[1]
Infusion (iv)/hour	157.15	130.01	206.05	gamma	[1]
BSC/cycle	1447.79	1164.03	1731.55	gamma	[1]
Palliative care/patient	21603.00	17282.40	25923.60	gamma	[1]
**Costs of serious TRAEs, $/cycle**					
Neutrophil count decreased	13656.00	10924.80	16387.20	gamma	[1]
White blood cell count decreased	13105.00	10484.00	15726.00	gamma	[1]
Platelet count decreased	13105.00	10484.00	15726.00	gamma	[1]
Anemia	7941.00	6352.80	9529.20	gamma	[1]

**Costs from Chinese healthcare sector perspective**					
**Costs of drugs, $/cycle**					
Adebrelimab (600mg)	1382.82	1106.26	1659.39	gamma	
Irinotecan (40 mg)	62.04	4.36	152.27	gamma	[1]
Cisplatin (30 mg)	3.32	2.80	4.09	gamma	[1]
Carboplatin (100 mg)	8.13	8.13	8.65	gamma	[1]
Etoposide (100 mg)	2.64	1.14	46.27	gamma	[1]
**Costs of administration**					
Laboratory/time	92.99	71.16	138.94	gamma	[1]
Imaging/time	989.47	638.60	1080.71	gamma	[1]
Infusion (iv)/hour	1.68	0.98	1.94	gamma	[1]
Best supportive care/cycle	345.60	95.10	952.50	gamma	[1]
Palliative care/patient	1460.30	1055.30	2085.70	gamma	[1]
**Costs of serious TRAEs, $/cycle**					
Neutrophil count decreased	115.01	51.11	357.80	gamma	[1]
White blood cell count decreased	115.01	51.11	357.80	gamma	[1]
Platelet count decreased	1505.92	1240.17	1771.67	gamma	[1]
Anemia	138.75	106.73	160.10	gamma	[1]
**Utility**					
**PB-utility**					

PFS	0.70	0.63	0.78	beta	[2]
PD	0.60	0.54	0.66	beta	[2]
**TTD-utility**					
> 10 cycles before death on treatment	0.73	0.71	0.74	beta	[3]
> 10 cycles before death off treatment	0.75	0.66	0.83	beta	[3]
> 5 cycles ≤ 10 cycles before death off treatment	0.70	0.62	0.77	beta	[3]
> 2 cycles ≤ 5 cycles before death off treatment	0.53	0.44	0.62	beta	[3]
≤ 2 cycles before death off treatment	0.33	0.22	0.42	beta	[3]
**Disutility of serious TRAEs**					
Neutrophil count decreased	0.20	0.14	0.26	beta	[4]
White blood cell count decreased	0.20	0.14	0.26	beta	[4]
Platelet count decreased	0.05	0.04	0.07	beta	[3]
Anemia	0.07	0.05	0.09	beta	[4]
**Risk of TRAEs**					
**Adebrelimab group**					
Neutrophil count decreased	0.76	0.61	0.91	beta	[5]
White blood cell count decreased	0.46	0.37	0.55	beta	[5]
Platelet count decreased	0.38	0.30	0.46	beta	[5]
Anemia	0.28	0.22	0.34	beta	[5]

**Chemotherapy group**					
Neutrophil count decreased	0.75	0.60	0.90	beta	[5]
White blood cell count decreased	0.38	0.30	0.46	beta	[5]
Platelet count decreased	0.34	0.27	0.41	beta	[5]
Anemia	0.28	0.22	0.34	beta	[5]
**Proportions of subsequent treatment**					
**Adebrelimab group**					
BSC	0.39	0.31	0.47	beta	[5]
Irinotecan	0.26	0.21	0.31	beta	[5]
Cisplatin	0.14	0.11	0.17	beta	[5]
**Chemotherapy group**					
BSC	0.26	0.21	0.31	beta	[5]
Irinotecan	0.38	0.30	0.46	beta	[5]
Cisplatin	0.18	0.14	0.22	beta	[5]
Carboplatin	0.10	0.08	0.12	beta	[5]
Etoposide	0.11	0.09	0.13	beta	[5]
**Discount rate**					
US	0.03	0.00	0.08	beta	[6]
China	0.05	0.03	0.08	beta	[7]

TRAEs, treatment-related adverse events; iv: intravenous injection; PB-utility, progression-based utility; PFS, progression-free survival; PD, progressive disease; TTD-utility, time-to-death utility; BSC, best supportive care.

eTable 5 Results of base-case analysis for the PFS state

Analysis Perspective			Cost, $	LYs	QALYs	Incremental Costs, $	Incremental QALYs	ICER, $/QALY
China	Model 1	Chemotherapy group	8435.1	0.57	0.39			
		Adebrelimab group	24362.55	1.03	0.68	15927.45	0.3	53661.92
	Model 2	Chemotherapy group	8910.05	0.57	0.39			
		Adebrelimab group	39661.14	1.03	0.68	30751.09	0.3	103604.95
US	Model 1	Chemotherapy group	29993.98	0.57	0.39			
		Adebrelimab group	48412.89	1.03	0.7	18418.91	0.31	59858.96
	Model 2	Chemotherapy group	30230.05	0.57	0.39			
		Adebrelimab group	64442.43	1.03	0.7	34212.39	0.31	111185.63

LYs, life-years; QALY, quality-adjusted life year; ICER, Incremental cost-effectiveness ratio.

eTable 6 Results of scenario analysis based on TTD-utility

Analysis Perspective			Cost, $	QALYs	Incremental Costs, $	Incremental QALYs	ICER, $/QALY
	**Overall survival**						
China	Model 1	Chemotherapy group	19698.74	0.95			
		Adebrelimab group	34976.93	1.32	15278.19	0.38	40481.74
	Model 2	Chemotherapy group	20173.69	0.95			
		Adebrelimab group	50275.52	1.32	30101.83	0.38	79759.11
	**Progression-free survival**						

	Model 1	Chemotherapy group	8435.1	0.4			
		Adebrelimab group	24362.55	0.71	15927.45	0.31	51455
	Model 2	Chemotherapy group	8910.05	0.4			
		Adebrelimab group	39661.14	0.71	30751.09	0.31	99344.05
	**Overall survival**						
	**Model 1**	Chemotherapy group	1267185.73	0.96			
		Adebrelimab group	1083752.59	1.35	-183433.14	0.39	
	**Model 2**	Chemotherapy group	1267421.8	0.96			
**US**		Adebrelimab group	1099782.13	1.35	-167639.66	0.39	
	**Progression-free survival**						
	**Model 1**	Chemotherapy group	29993.98	0.41			
		Adebrelimab group	48412.89	0.73	18418.91	0.32	57397.24
	**Model 2**	Chemotherapy group	30230.05	0.41			
		Adebrelimab group	64442.43	0.73	34212.39	0.32	106613.08

TTD-utility, time-to-death utility; QALY, quality-adjusted life year; ICER, incremental cost-effectiveness ratio.

eTable 7 Results of scenario analysis based on PAP

Analysis Perspective			Cost, $	QALYs	Incremental Costs, $	Incremental QALYs	ICER, $/QALY
	**Overall survival**						
**PB-utility**	**Model 1**	Chemotherapy group	19698.74	0.82			
		Adebrelimab group	26342.97	1.17	6644.23	0.35	18893.47
	**Model 2**	Chemotherapy group	20173.69	0.82			

		Adebrelimab group	26817.91	1.17	6644.23	0.35	18893.47
	**Progression-free survival**						
	**Model 1**	Chemotherapy group	8435.1	0.39			
		Adebrelimab group	15728.59	0.68	7293.49	0.3	24572.83
	**Model 2**	Chemotherapy group	8910.05	0.39			
		Adebrelimab group	16203.53	0.68	7293.49	0.3	24572.83
**TTD-utility**	**Overall survival**						
	**Model 1**	Chemotherapy group	19698.74	0.95			
		Adebrelimab group	26342.97	1.32	6644.23	0.38	17604.79
	**Model 2**	Chemotherapy group	20173.69	0.95			
		Adebrelimab group	26817.91	1.32	6644.23	0.38	17604.79
	**Progression-free survival**						
	**Model 1**	Chemotherapy group	8435.1	0.4			
		Adebrelimab group	15728.59	0.71	7293.49	0.31	23562.34
	**Model 2**	Chemotherapy group	8910.05	0.4			
		Adebrelimab group	16203.53	0.71	7293.49	0.31	23562.34

PAP, patient assistance program; QALY, quality-adjusted life year; ICER, incremental cost-effectiveness ratio; PB-utility, progression-based utility; TTD-utility, time-to-death utility.

eTable 8 Dose and average costs per cycle of PD-L1/PD-1 drugs simultaneously approved in the US and China for the treatment of lung cancer

**Drugs**	**Nivolumab**	**Pembrolizumab**	**Atezolizumab**	**durvalumab**
**Dose per cycle, mg/cycle**	360	200	200	1500

**US**	**Average costs per 100mg, $/100mg**	3597.10	6410.12	1009.48	933.58
	**Average costs per cycle, $/cycle**	12949.56	12820.24	2018.96	14003.70
**China**	**Average costs per 100mg, $/100mg**	1507.80	2608.15	7957.30	3515.75
	**Average costs per cycle, $/cycle**	5428.08	5216.30	15914.60	52736.25

PD-L1/PD-1, programmed cell death-1/programmed cell death receptor ligand-1.

A1

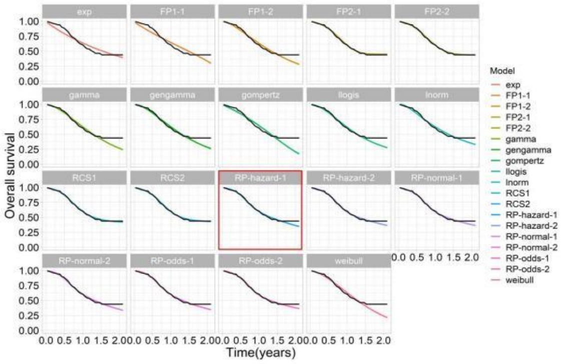

B1

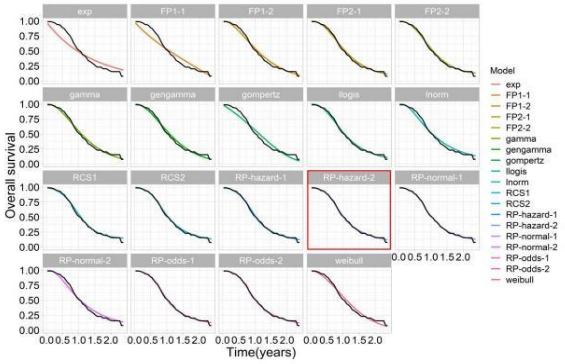

C1

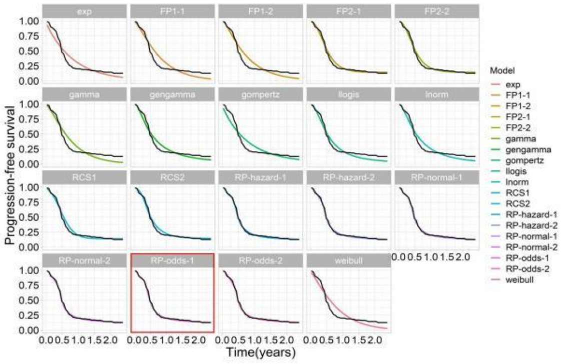

D1

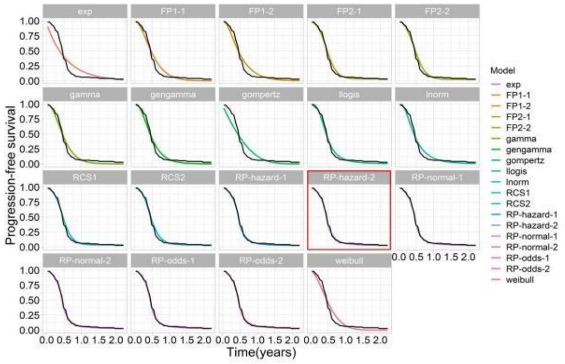

A2

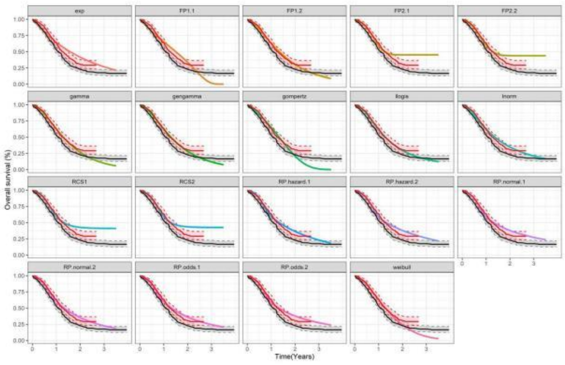

B2

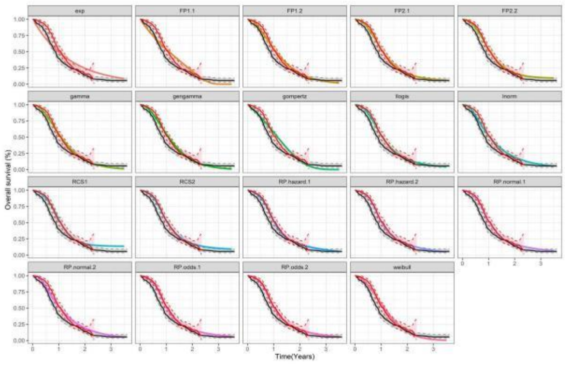

C2

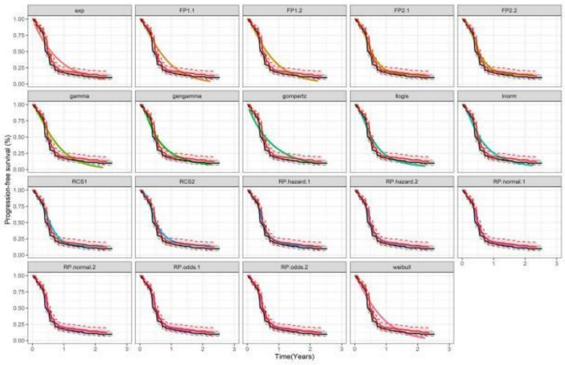

D2

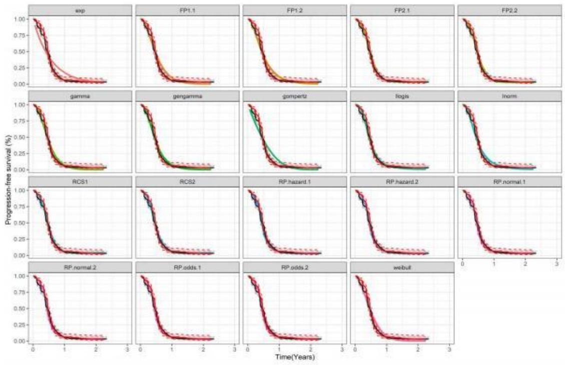

eFigure 1 Survival plots for goodness-of-fit and external validatio of parametric survival models. (A_1) Survival plots for goodness-of-fit of parametric survival models for K-M curve of OS in patients with adebrelimab plus chemotherapy, (B_1) Survival plots for goodness-of-fit of parametric survival models for K-M curve of OS in patients with chemotherapy, (C_1) Survival plots for goodness-of-fit of parametric survival models for K-M curve of PFS in patients with adebrelimab plus chemotherapy, (D_1) Survival plots for goodness-of-fit of parametric survival models for K-M curve of PFS in patients with chemotherapy; black lines showed the original K-M curves, the red square was the best model. (A_2) Survival plots for external validatio of parametric survival models for K-M curve of OS in patients with adebrelimab plus chemotherapy, (B_2) Survival plots for external validatio of parametric survival models for K-M curve of OS in patients with chemotherapy, (C_2) Survival plots for external validatio of parametric survival models for K-M curve of PFS in patients with adebrelimab plus chemotherapy, (D_2) Survival plots for external validatio of parametric survival models for K-M curve of PFS in patients with chemotherapy; black lines showed the original K-M curves, red lines showed the external K-M data, dashed lines represented the 95%CI, lines in other colors represented the modeled data.

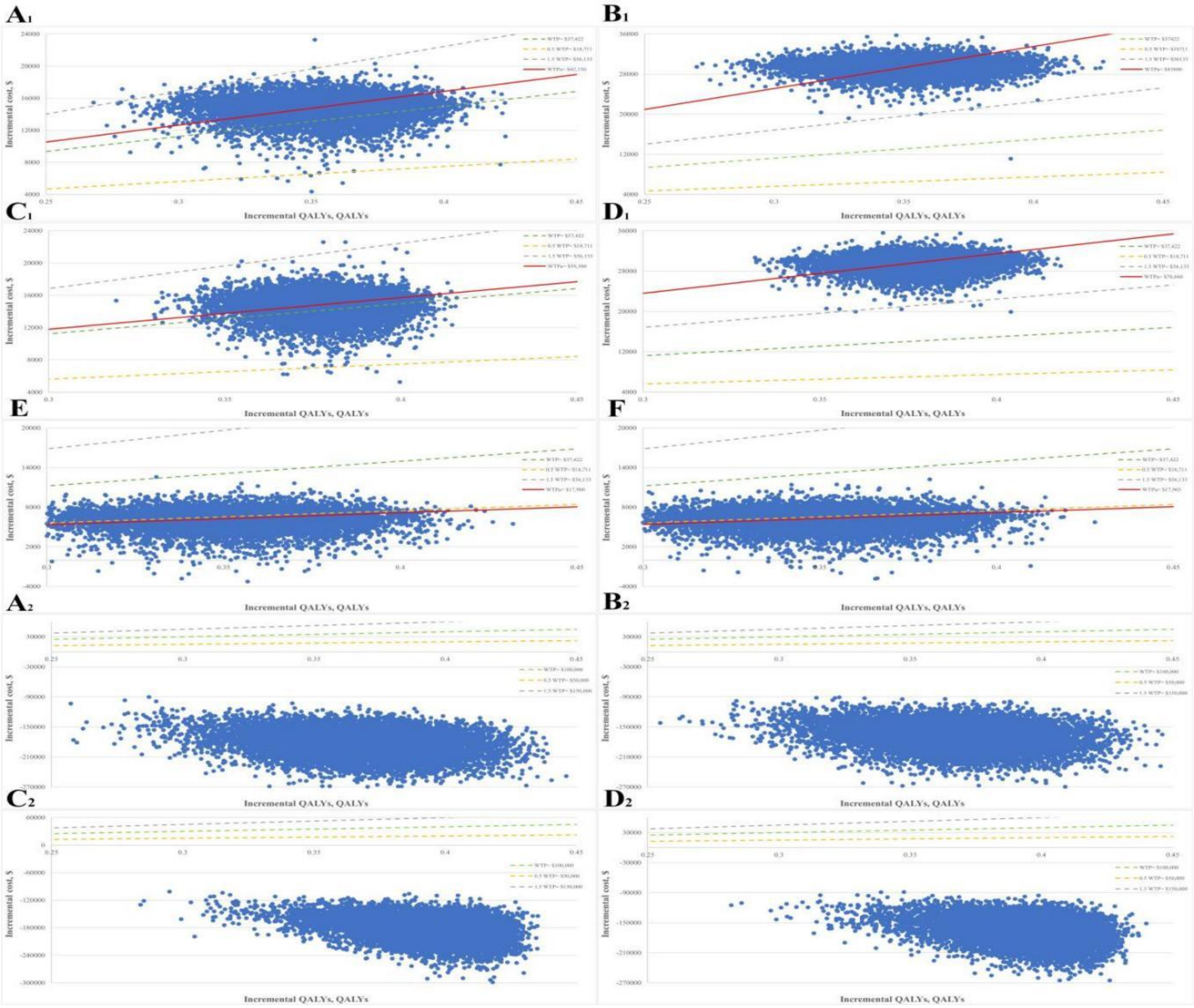

eFigure 2 Scatter plots of incremental QALYs and costs in the PSA at $1382.82 of adebrelimab price. (A_1) Results of PSA in model 1 for base-case from Chinese perspective, (B_1) Results of PSA in model 2 for base-case from Chinese perspective, (C_1) Results of PSA in model 1 for scenario 1 from Chinese perspective, (D_1) Results of PSA in model 2 for scenario 1 from Chinese perspective, (E) Results of PSA in model 1 for scenario 2 from Chinese perspective, (F) Results of OWSA in model 2 for scenario 2 from Chinese perspective. (A_2) Results of PSA in model 1 for base-case from the US perspective, (B_2) Results of PSA in model 2 for base-case from the US perspective, (C_2) Results of PSA in model 1 for scenario 1 from the US perspective, (D_2) Results of PSA in model 2 for scenario 1 from the US perspective.

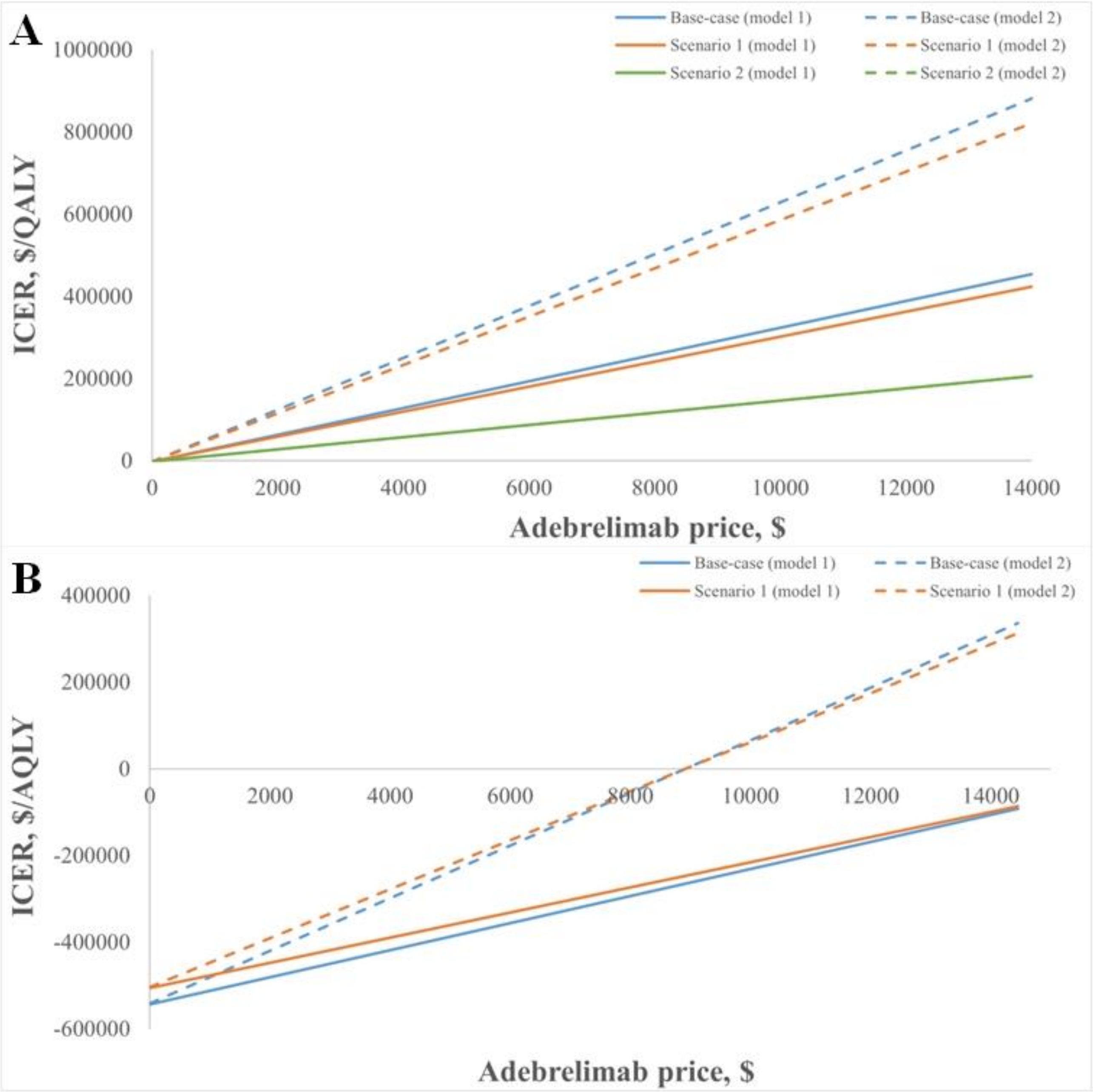

eFigure 3 The relationship between adebrelimab price and ICER. (A) The relationship between adebrelimab price and ICER from Chinese perspective, (B) The relationship between adebrelimab price and ICER from the US perspective.

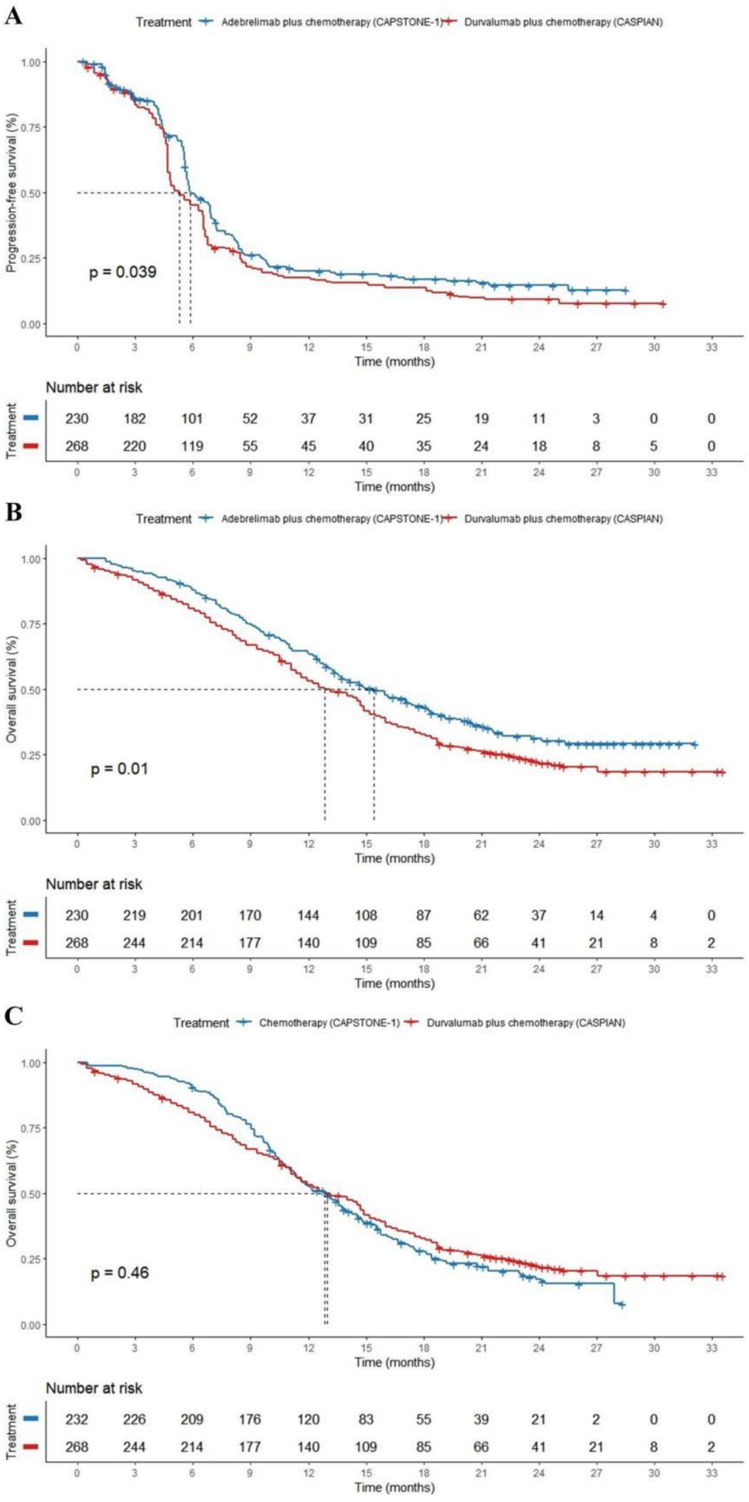

eFigure 4 The K-M curves of OS and PFS from the CAPSTONE-1 and CASPIAN trials. (A) The K-M curves of OS between patients with adebrelimab plus chemotherapy in the CAPSTONE-1 trial and patients with durvalumab plus chemotherapy in the CASPIAN trial, (B) The K-M curves of PFS between patients with adebrelimab plus chemotherapy in the CAPSTONE-1 trial and patients with durvalumab plus chemotherapy in the CASPIAN trial, (C) The K-M curves of PFS between patients with chemotherapy in the CAPSTONE-1 trial and patients with durvalumab plus chemotherapy in the CASPIAN trial.

References

1. Shao, T., et al., *Serplulimab Plus Chemotherapy vs Chemotherapy for Treatment of US and Chinese Patients with Extensive-Stage Small-Cell Lung Cancer: A Cost-Effectiveness Analysis to Inform Drug Pricing.* BioDrugs, 2023.
2. Vedadi, A., et al., *The impact of symptoms and comorbidity on health utility scores and health-related quality of life in small cell lung cancer using real world data.* Qual Life Res, 2021. **30**(2): p. 445-454.
3. Excellence, N.I.f.H.a.C. *Atezolizumab with carboplatin and etoposide for untreated extensive-stage small-cell lung cancer*. Available from: https://www.nice.org.uk/guidance/ta638/evidence.
4. Nafees, B., et al., *Health state utilities in non-small cell lung cancer: An international study.* Asia Pac J Clin Oncol, 2017. **13**(5): p. e195-e203.
5. Wang, J., et al., *Adebrelimab or placebo plus carboplatin and etoposide as first-line treatment for extensive-stage small-cell lung cancer (CAPSTONE-1): a multicentre, randomised, double-blind, placebo-controlled, phase 3 trial.* Lancet Oncol, 2022. **23**(6): p. 739-747.
6. Su, D., B. Wu, and L. Shi, *Cost-effectiveness of Atezolizumab Plus Bevacizumab vs Sorafenib as First-Line Treatment of Unresectable Hepatocellular Carcinoma.* JAMA Netw Open, 2021. **4**(2): p. e210037.
7. Yue, X., et al., *Current Development and Practice of Pharmacoeconomic Evaluation Guidelines for Universal Health Coverage in China.* Value Health Reg Issues, 2021. **24**: p. 1-5.
